# Effects of Assist-As-Needed Upper Extremity Robotic Therapy after Incomplete Spinal Cord Injury: A Parallel-Group Controlled Trial

**DOI:** 10.3389/fnbot.2017.00026

**Published:** 2017-06-13

**Authors:** John Michael Frullo, Jared Elinger, Ali Utku Pehlivan, Kyle Fitle, Kathryn Nedley, Gerard E. Francisco, Fabrizio Sergi, Marcia K. O’Malley

**Affiliations:** ^1^Department of Mechanical Engineering, Rice University, Houston, TX, United States; ^2^TIRR Memorial Hermann, Houston, TX, United States; ^3^Department of Physical Medicine and Rehabilitation, University of Texas Health Science Center, Houston, TX, United States; ^4^Department of Biomedical Engineering, University of Delaware, Newark, DE, United States

**Keywords:** robot-aided rehabilitation, assist-as-needed therapy, motor learning, incomplete spinal cord injury, adaptive control

## Abstract

**Background:**

Robotic rehabilitation of the upper limb following neurological injury has been supported through several large clinical studies for individuals with chronic stroke. The application of robotic rehabilitation to the treatment of other neurological injuries is less developed, despite indications that strategies successful for restoration of motor capability following stroke may benefit individuals with incomplete spinal cord injury (SCI) as well. Although recent studies suggest that robot-aided rehabilitation might be beneficial after incomplete SCI, it is still unclear what type of robot-aided intervention contributes to motor recovery.

**Methods:**

We developed a novel assist-as-needed (AAN) robotic controller to adjust challenge and robotic assistance continuously during rehabilitation therapy delivered via an upper extremity exoskeleton, the MAHI Exo-II, to train independent elbow and wrist joint movements. We further enrolled seventeen patients with incomplete spinal cord injury (AIS C and D levels) in a parallel-group balanced controlled trial to test the efficacy of the AAN controller, compared to a subject-triggered (ST) controller that does not adjust assistance or challenge levels continuously during therapy. The conducted study is a stage two, development-of-concept pilot study.

**Results:**

We validated the AAN controller in its capability of modulating assistance and challenge during therapy via analysis of longitudinal robotic metrics. For the selected primary outcome measure, the pre–post difference in ARAT score, no statistically significant change was measured in either group of subjects. Ancillary analysis of secondary outcome measures obtained via robotic testing indicates gradual improvement in movement quality during the therapy program in both groups, with the AAN controller affording greater increases in movement quality over the ST controller.

**Conclusion:**

The present study demonstrates feasibility of subject-adaptive robotic therapy after incomplete spinal cord injury, but does not demonstrate gains in arm function occurring as a result of the robot-assisted rehabilitation program, nor differential gains obtained as a result of the developed AAN controller. Further research is warranted to better quantify the recovery potential provided by AAN control strategies for robotic rehabilitation of the upper limb following incomplete SCI.

**ClinicalTrials.gov registration number**: NCT02803255.

## 1 Introduction

The annual incidence of spinal cord injury (SCI), not including those who die at the scene of injury, is approximately 40 cases per million in the United States or approximately 12,000 new cases each year (National Spinal Cord Injury Statistical Center, 2012). SCI primarily affects young adults, with an average age at injury of 41 years and average lifetime costs exceeding a million dollars per subject in the U.S. Neurologically induced deficits in motor function are common following complete and incomplete tetraplegia and result from partial or complete paralysis of muscles. Complete paralysis results in the inability to activate muscles below the level of injury. Partial paralysis occurs from disruption to some but not all neural pathways innervating muscles. 40.8% of survivors are subject to incomplete tetraplegia, followed by 21.6% of survivors categorized as complete paraplegia, 21.4% categorized as incomplete paraplegia and 15.8% as complete tetraplegia. As a result of the injury, two-thirds of SCI survivors are left with some functional deficit to the upper extremity, which contributes to reduced independence in activities of daily living. Improvements in arm and hand function may increase independence in self-care, increase engagement in social activities, decrease caregiver burden, and improve quality of life.

It has recently been suggested that repetitive movement exercise can support recovery after SCI by enhancing some form of plasticity intrinsic in the central nervous system (Raineteau and Schwab, 2001; Cai et al., 2006; Lynskey et al., 2008; Onifer et al., 2011). Given the association between treatment intensity and potential for motor recovery, robotic technologies have been used to automate repetitive movement exercise after incomplete spinal cord injury lesions. Most of the existing research efforts in SCI rehabilitation have addressed gait training (Hornby et al., 2005; Shin et al., 2014), whereas robotic training of upper extremity function after SCI is much less developed, with only a few case studies presented so far (Yozbatiran et al., 2012; Cortes et al., 2013). Such case studies demonstrated feasibility of robotic training after incomplete SCI, but could not demonstrate statistically significant gains in motor function achieved via the intervention. This is in contrast to the field of robot-assisted stroke rehabilitation, where large-scale trials have shown that robotic intervention can safely and effectively induce recovery in upper extremity motor function after stroke (Lo et al., 2010; Klamroth-Marganska et al., 2014).

Robots are capable of automating movement therapy according to a wide variety of programmable control modes. Numerous investigators applied dynamic systems and control theory to formulate robot controllers suitable for poststroke rehabilitation (Marchal Crespo and Reinkensmeyer, 2009). Different controller implementations have been proposed, each focusing on a specific aspect of robotic therapy, such as assisting movements only if they are not properly timed (Krebs et al., 2003), modulating error by perturbing movements during therapy (Patton et al., 2005), guiding joints along predetermined, time-independent trajectories (Banala et al., 2009), and combining real-time subject force estimation with adaptation of feedforward (Wolbrecht et al., 2008) or feedback and feedforward force assistance (Pehlivan et al., 2015). Although some details differ with each implementation, the rationale behind development of a specific control mode for rehabilitation therapy is mostly inspired by prior human subject studies (Lewis and Byblow, 2002; Hogan et al., 2006), suggesting that intensive therapy delivered by robotic interaction modes aimed at maximizing the active participation of the subject would be a catalyst for the process of neural plasticity underlying motor recovery after stroke (Mehrholz et al., 2013).

As robot-aided recovery after incomplete SCI is at a relatively less mature stage than that of stroke, such reference human subject studies are not yet present. Despite studies on animal models suggesting that rehabilitation should leverage plasticity through stimuli similar to those tested for stroke rehabilitation (Cai et al., 2006; van den Brand et al., 2012), optimal treatment regimes for robot-aided rehabilitation are far from having been identified. Especially in rehabilitation after incomplete SCI, a field still much in its infancy, early-stage trials should be aimed at giving inputs for further refinement of robot-assisted therapeutic protocols.

Such inputs can be provided by parallel-group controlled trials (PGCT). In a PGCT, the specific effect of a treatment modality is assessed by measuring a variable (outcome measure) in a group undergoing treatment, and comparing the outcome measure with the one obtained in a parallel group, where an alternative treatment is delivered. If a clinical study intends to evaluate the specific effects of a novel controller, it should compare the effects of this controller not to the absence of rehabilitation, but instead to a different, *standard of care* form of rehabilitation. Through this methodology, it would be possible to isolate the differential effects of the investigated treatment and control for a wide variety of other factors that might have an effect on recovery. In fields where there is an established standard of care, this is usually done by comparing the results achievable through a new treatment with literature data. However, application of this approach is made difficult by the fact that there are no robust reference data for robot-assisted upper extremity training after SCI. In general, testing the efficacy of rehabilitation paradigms is complicated by the large variability of subject populations, both in terms of baseline motor function and in terms of pre vs. post improvement of motor function. High variability of baseline and improvement variables leads to demand for multicenter studies, especially in SCI rehabilitation, where low prevalence provides challenges even in large cities.^1^ Instead, large-scale clinical studies such as multicenter studies are not appropriate for early stage trials where it is desired to test a particular aspect of a therapeutic protocol (e.g., the robot control mode), whose validity can be tested for later inclusion in larger phase II or phase III randomized controlled trials, following the framework for staging motor intervention studies proposed in Dobkin (2008). From the consideration above, it is indeed not a surprise that most of the large-scale clinical investigations of rehabilitation robotics could only test the feasibility of robotic rehabilitation and could not go more in depth assessing the differential effects of a specific control mode (Lo, 2012).

**Figure 1:**
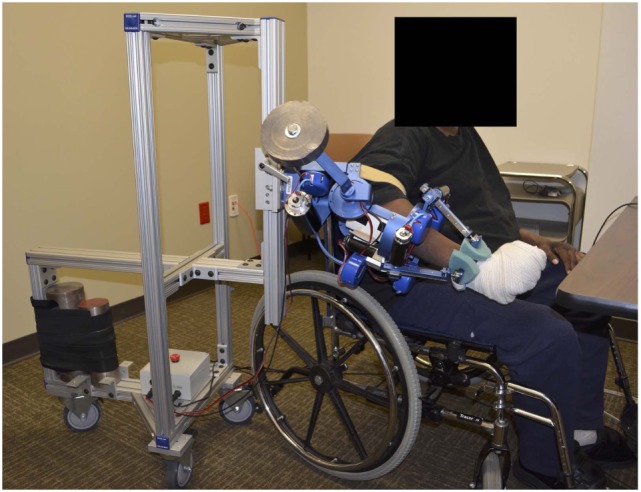
Subject using the MAHI Exo-II robotic upper limb exoskeleton.

In this study, we evaluate the effect of two different interactive schemes implemented on the MAHI Exo-II robotic upper limb exoskeleton (Figure 1), on therapy outcomes in a population of subjects with incomplete spinal cord injury. We hypothesized that a subject-adaptive controller, capable of continuously adapting the levels of assistance and challenge provided during movement-based rehabilitation therapy, enabled achievement of higher gains in arm function after chronic incomplete spinal cord injury, compared to a non-adaptive, subject-triggered position controller. This study serves the dual purpose of assessing the potential of subject-adaptive interaction control schemes for robot-aided therapy after incomplete spinal cord injury and of guiding the development of more sophisticated interaction controllers for upper extremity rehabilitation therapy.

## 2 Materials and Methods

### 2.1 Study Design

The study followed a PGCT design, where subjects were assigned to two different robotic interventions, namely, the assist-as-needed (AAN) and the subject-triggered (ST) controller, detailed in the following sections. The null hypothesis tested in this study was that the change in motor function for subjects exposed to the AAN paradigm would be the same as the one obtained through the ST paradigm.

Participants with cervical motor incomplete SCI were assigned to either the AAN group or to the ST group. Inclusion criteria were age (comprised between 18 and 75 years), diagnosis of chronic incomplete SCI affecting upper extremity function (American Spinal Injury Association (ASIA) Impairment Scale (AIS) C-D levels, with the injury occurring at least 6 months prior to enrollment), while exclusion criteria were prior participation in robotic rehabilitation studies for the upper arm, any planned alteration in medication for muscle tone for the duration of the study, arthritis, excessive shoulder pain, joint contracture or excessive muscle tone (Modified Ashworth Scale >3). Although the inclusion and exclusion criteria did not target specific locations of injury, the requirement “incomplete SCI affecting upper extremity function” resulted in admitted participants with lesion levels comprised between C3 and C8 (Table 1).

**Table 1:** Characteristics of recruited subjects.

Subject	Group	Age range	Time since injury (years)	Baseline ARAT	AIS
R01	ST	61–65	2	53	C3
R02	ST	46–50	26	47	C6
R03	ST	46–50	14	19	C5–6
R04	ST	56–60	3	16	C3
R05^a^	AAN	21–25	2	35	C7–8
R06^a^	AAN	21–25	1		
R07	AAN	61–65	12	41	C6–7
R08	AAN	36–40	23	11	C4
R09^b^	AAN	46–50	2	45	C4
R10	AAN	51–55	8	45	C4
R11	ST	46–50	16	7	C4
R12	AAN	46–50	16	21	C4
R13	AAN	56–60	37	20	C3
R14^a^	ST	26–30	4	18	C3–4
R15	AAN	66–70	2	3	C4
R16	ST	46–50	36	21	C4
R17	ST	51–55	26	6	C4–5

*^a^R05, R06, and R14 dropped during the course of the study*.

*^b^R09 dropped after the posttreatment session*.

The study was designed to test for significant differences between the change in functional measures obtained through AAN control and the one obtained through ST control. Thus, a 2-sided type I error of 0.05 was used for the primary treatment comparison. Sample size was calculated for a 2-sample *t*-test to detect a mean difference of 3 points in the primary outcome measure, i.e., the ARAT scale (see outcome measures section below), with 90% power, assuming a common standard deviation of 2 points in the ARAT score (calculated from the results of a previous study with 8 SCI survivors undergoing resistance training (Fitle et al., 2015)), and a loss rate of 20%.^2^ A sample size of 24 admitted participants was required to detect the hypothesized 3-point difference in the two treatment groups, resulting in a final population of 10 subjects per group completing the study (20 subjects in total), given the 20% loss rate expected. When merged together in a comparison of the overall effects of both rehabilitation modes, the resulting 1-sample *t*-test with the 20 participants has 90% power to test significant differences in the increase in ARAT score of 1.5, with a type I error rate of 0.05.

### 2.2 Participants

Study participants were recruited by referral from therapists at a partnering institution (TIRR Memorial Hermann in Houston, TX, USA) or were enrolled after they contacted the PI as a result of flyers placed in several rehabilitation clinics in the Houston, TX, USA, area. In total, 37 people were contacted and screened. 17 subjects (46%) were enrolled in the study, with the remaining 20 (54%) either failing to comply with the inclusion criteria or simply showing lack of interest in the study.

This study was reviewed and approved by the Institutional Review Boards (IRB) of Rice University and our clinical collaborators’ institutions (Rice University IRB 654451, UT Health/TIRR Memorial Hermann IRB HSC-GEN-13-0315), with written informed consent from all subjects. All subjects gave written informed consent in accordance with the Declaration of Helsinki. This study has been retrospectively registered on Clinicaltrials.gov, registration number: NCT02803255.

Three subjects (18% of the enrolled group, similar to the 20% loss rate expected) dropped out during therapy due to logistical reasons, and one subject did not return for the post-treatment evaluation (this subject is considered in the group analyses because he only missed the two-week and two-month follow-up assessments). For the 14 who completed the study, 12 were male (86%). We did not collect race/ethnicity information. The group average age was 53.5 years old, the average time since injury was 16 years, and the average baseline ARAT score was 25. See Table 1 for specific subject information.

Assignment of subjects to a specific group was conducted using the method for covariate minimization described in our preliminary work (Sergi et al., 2015), which sought to minimize the imbalance in the two groups of factors potentially associated with future gains in motor function. For this study, our subject assignment algorithm sought to minimize the imbalance of age and baseline ARAT score. After the first four subjects were assigned to the ST group, the group assignment method provided balanced groups in terms of the two prognostic variables (ARAT and age), (Δ*_ARAT_* = 1.8 points, σ*_ARAT_* = 17.24 points, Δ*_age_* = 1.6 years, σ*_age_* = 7.2 years), or better than 76% of the entire set of possible random assignments to both groups, as demonstrated by a *post hoc* analysis based on the systematic assessment of all possible permutations of enrolled subjects.

### 2.3 Protocol

**Figure 2:**
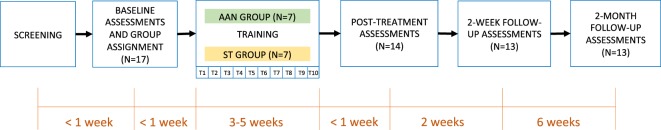
Flow diagram describing progression of subjects through the study.

Each subject participated in a total of fifteen visits. The first two visits involved screening for inclusion and exclusion criteria and a baseline assessment on primary and secondary outcome measures, in addition to the ASIA upper extremity scale to verify the diagnosis. Within one week after the last baseline visit, subjects started a program of robotic training, in ten 90-min long sessions, spread over a period of three to four weeks (the number of visits per week ranged between 1 and 3, depending on subject availability and scheduling constraints for baseline and follow-up visits). After the last training session, three posttreatment clinical assessment sessions (one week, two weeks, and two months after treatment) were completed with the therapist. The progression of subjects through the study is presented in Figure 2.

Group assignment was implemented after the first screening session based on the result of the pre-therapy ARAT test. Subject assignment was undisclosed to the occupational therapist performing the evaluations (KN), who did not participate in any of the therapy sessions, enabling complete blinding of the study.

At the beginning of each robotic training session, subjects underwent an evaluation session, then robotic training, which took the form of *p* repetitions of single-DOF movements, with *p* adapted to result in sessions of the prescribed duration (90 min total). In evaluation sessions, the subjects’ range of motion (ROM) was calculated by asking the subjects to move a given joint in both directions to the maximum level that they considered comfortable, and recording the maximum and minimum values angles using the MAHI Exo-II encoders. During evaluation sessions, the MAHI Exo-II was unpowered, opposing minimal resistance to motion due to its backdrivable design. Evaluation sessions were based on point-to-point movements from a center target (placed at the middle point between the two extremes calculated before) to the periphery targets defined in the ROM procedure. Although the MAHI Exo-II allows training of complex movements combining both elbow and wrist joints, we chose to train subjects in uni-dimensional tasks based on recent literature demonstrating that training complex movements does not lead to a greater improvement in motor function in stroke patients (Milot et al., 2013).

During training sessions, subjects similarly underwent repeated point-to-point movements per DOF. The number of repetitions was initially specified as the final value in the previous session, and then increased based on the availability of time. Training sessions lasted 90 min, with setup taking approximately 5 min per subject.

For patients in the AAN controller group, both assistance and timing parameters estimated from the previous sessions were retained as an initial guess in the subject-adaptive therapy mode, whereas for patients in the ST controller group, the therapist manually set the challenge parameters (force threshold, *F_th_*, and time allowed for a movement, *T_ST_*) on a session-by-session basis, based on the subject’s qualitative assessment of fatigue over the course of the session and the 90-min duration constraint.

### 2.4 Exoskeleton and Control Modes

During therapy, subjects interacted with the MAHI Exo-II, a four degree-of-freedom (DOF) exoskeleton used for isolated rehabilitation of the elbow (flexion/extension) and the wrist (pronation/supination—PS, radial-ulnar deviation—RUD, flexion/extension—FE). Details on the mechanical design of the robot are included in prior work (Pehlivan et al., 2011). The robot, shown in Figure 1, is a unilateral upper extremity exoskeleton supported by a moving aluminum frame that allows an easy adjustment to fit the arm of subjects sitting on a chair. The exoskeleton has four degrees of freedom actuated by DC motors and cable transmissions and is connected to the subjects’ arm via thermoplastic cuffs that connect to the subject upper arm, and forearm, with both contacts secured by velcro straps. The wrist component of the exoskeleton terminates with a handle, which is grasped by the subject (or is strapped to the subject’s hand in case of individuals with limited grasping capabilities), which allows the device to track and assist the wrist rotation angles after solving the forward kinematics of the Revolute Prismatic Spherical wrist component (RiceWrist) (Gupta et al., 2008; Erwin et al., 2015, 2016). Motion of the upper arm is prevented by soft contacts via velcro straps; however, the subject torso was not constrained to maximize subject comfort in the intensive therapy program. Similarly, we found that by using soft constraints and velcro straps, subjects could operate comfortably the robot without requiring highly accurate alignment of the robotic degrees of freedom to the subject joints. The time required for fitting a new subject in the robot never exceeded 15 min, with setup for subsequent visits being considerably shorter. The MAHI Exo-II was programmed via two different control modes, the assist-as-needed (AAN) controller, and the subject-triggered (ST) controller, described in detail in the following sections.

#### 2.4.1 Assist-As-Needed Controller

**Figure 3:**
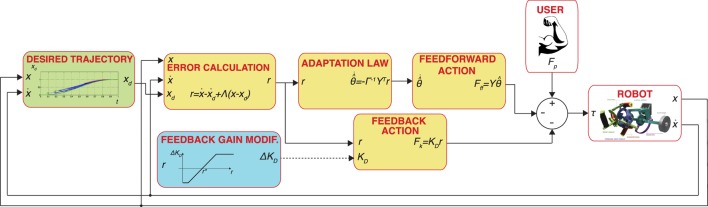
Block diagram of the AAN controller implemented in this paper. Blocks with a yellow background include components of the adaptive controller (Slotine and Li, 1987). The dashed line refers to a discontinuous update of signal variables, i.e., the feedback gain is changed on a task-by-task basis.

For the AAN controller (Figure 3), we adapted the controller proposed in the study by Pehlivan et al. (2015), which consists of three main components: subject force estimation, feedback gain modification, and online trajectory recalculation. The subject ability estimation algorithm employed in this study is a model-based estimator based on the adaptive controller (Slotine and Li, 1987). The controller is based on the general form of the dynamic equations of a human-interacting manipulator in the task space (defined by independent generalized coordinates *x*):

(1) $$M\left(x\right)ẍ+C\left(x,ẋ\right)ẋ+G\left(x\right)=F_{r}+F_{p},$$

where *M* is the manipulator inertia matrix, *C* is the matrix of Coriolis/centrifugal terms, *G* is the gravity vector, *F_r_* = *J*^−^*^T^F_a_* is the vector of equivalent end-effector generalized forces applied by the actuators, and *F_p_* is the vector of end-effector generalized forces applied by the subject. Different from the study by Slotine and Li (1987), our controller neglects the inertial, Coriolis, and centrifugal terms and applies an assistance force/torque defined as:

(2) $$F_{r}=Ĝ\left(x\right)-\;{\hat{F}}_{p}-K_{D}r,$$

where Ĝ(x) and ${\hat{F}}_{p}$ are respectively estimates of the gravitational term and patient-applied force, and *K_D_r* is a feedback corrective term, based on the sliding variable

(3) $$r=\dot{\tilde{x}}+\Lambda \tilde{x}=\left(ẋ-x_{d}\right)+\Lambda \left(x-x_{d}\right).$$

In our previous work, we used a linear parameterization based on the regression matrix *Y* (*x*) and unknown parameters *θ*:

(4) $$Y\left(x\right)\hat{\theta }=Ĝ\left(x\right)-{\hat{F}}_{p},$$

and the adaptation law

(5) $$\dot{\tilde{\theta }}=-\Gamma^{-1}Y{\left(x\right)}^{T}r$$

where Γ is an *n* × *n* constant, positive definite, symmetric matrix; *Y* is a matrix of regressors which contains known functions of *x*, based on a set of Gaussian radial basis functions (RBFs) to approximate the position dependence of terms in the right side of equation (4). For this study, considering that an impaired subject might have different levels of disability on their agonist and antagonist muscles, we extended our previous formulation by introducing direction dependency on the regressor matrix *Y*  = *Y* (*x*, ẋ). As in the study by Pehlivan et al. (2015), we use RBFs as known functions included in the regressor matrix, but we doubled the set of RBFs for each DOF to account for direction dependence (i.e., we compute different sets of RBFs for positive and negative derivatives of the task space controlled variables for each DOF).

We finally introduced a feedback gain modification logic, a component required for modulating the amount of motion assistance in a performance-adaptive way. For this study, we discretely updated the trial-to-trial change of the feedback gain, Δ*K_D_*, based on the measured error in the previous task. Δ*K_D_* is defined as

(6) $$\Delta K_{D}=\Delta K_{D,\text{max}}\frac{\left(r_{\text{avg}}-r^{*}\right)}{\left(r^{*}-r_{\text{min}}\right)},$$

where Δ*K_D,max_* is a scaling factor of the trial-by-trial change of the feedback gain, *r_avg_* is the average error for the previous task, and *r_min_* defines the slope of the gain update curve. The same gain update logic had been validated in a similar subject-adaptive controller, tested on healthy individuals, and presented in detail in the study by Pehlivan et al. (2016). With the gain update law shown in (6), we introduce an error characteristic term, r*, such that for errors below the threshold the feedback gain is increased, while for errors above the threshold the gain is decreased. With this formulation, we are able to account for the fact that even healthy subjects’ movements contain natural variability and providing force support to minimize error beyond such variability might be detrimental to motor learning (Shadmehr et al., 2010). Both the values of r* and *r_min_* were defined as a proportion of the amplitude of the subject range of motion, with values shown in Table 2.

**Table 2:** AAN controller parameters.

DOF	*r_min_* [%]	r* [%]	*K_D,in_* [Nms/deg]	*K_D,max_* [Nms/deg]	*T_in_* [s]
Elbow	0	0.5	0.5	2.89	2
Wrist PS	0.3	2.5	0.5	1	2
Wrist FE	0.06	10	0.33	0.25	2
Wrist RUD	0.06	10	0.3	0.25	2

**Figure 4:**
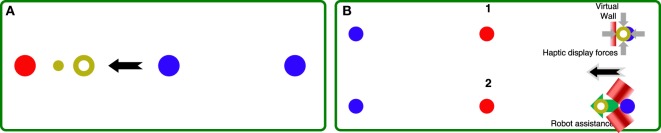
**(A)** GUI used in the AAN controller, during the online recalculation “off” phase. The red circle corresponds to the active target, the blue circles are the other targets (center and periphery). The current subject position is displayed with the yellow ring, while the ghost cursor is the smaller yellow cursor leading the subject in this center-to-periphery movement (black arrow). **(B)** Sequence of the two modes of the ST controller. (1) A virtual wall is implemented, and the force required to keep the desired position (blue circle) is continuously measured. When the force exceeds *F_th_*, the system switches to mode (2), where the robot implements position control toward the target (red circle).

The generation of the desired trajectory *x_d_*(*t*) for this controller is based on our previous work, validated on healthy subjects (Pehlivan et al., 2015). At the beginning of the movement, a nominal desired trajectory based on a physiological joint movement profile, and allocated time *T_end_* is defined. During the movement, a conditional trajectory recalculation (CTR) is implemented, so that when the position of the subject is ahead of the nominal desired trajectory, a new desired trajectory is computed as a piecewise polynomial function. For each recalculation, the parameter *T_end_* is reduced for the current movement by 1%, and the updated value of *T_end_* is kept for the next task. In an attempt to differentiate between intentional subject involvement and unintentional elastic return due to muscle stretching, the CTR is here enabled only if the subject is able to be ahead of the nominal desired trajectory in both center-to-periphery and following periphery-to-center directions for a percentage (10%) of the last movement when CTR was disabled. This helps guarantee active subject input because the elastic return of stretched muscles typically only aids movement from periphery-to-center. If the CTR is not activated for a given task, the algorithm will increase *T_end_* by 0.2 s until the subject is able to stay ahead of the desired trajectory. During the CTR “off” phase, a ghost cursor following the nominal desired trajectory is displayed to the subject in the GUI to motivate the subject to be ahead of the nominal trajectory (see Figure 4A). Since a lead-type error is not possible when the trajectory recalculation mode is switched on, the RBF amplitude estimates are mostly non-decreasing (in absolute value) in this condition, resulting in an overestimate of the feedforward assistance. To avoid this problem, the adaptation law in equation (5) is modified to include a first-order decay of the RBF amplitude estimates only when the error drops below the value *r_min_*.

The initial allotted time for all DOFs was 2 s, and the initial gains were defined as shown in Table 2 equally for all subjects, and then free to change as defined by the AAN algorithm. The AAN controller was implemented in Matlab/Simulink (The MathWorks, Inc.) and data acquisition at a sampling rate of 1 kHz was achieved using the soft real-time software QuaRC (Quanser Inc.). A command-line interface allowed specification of control parameters, such as joint gain values, allotted time, and number of repetitions for each section.

#### 2.4.2 Subject-Triggered Controller

The subject-triggered controller is implemented to require subjects to initiate therapeutic movements with the robot, then having the robot carry the passive limb through the desired trajectory. The controller is identical to one developed for upper-limb robotic rehabilitation following chronic stroke (Lum et al., 2002) and later implemented on the MAHI Exo-II rehabilitation robot (Gupta et al., 2008).

The ST controller is implemented as a two-state machine. In the first state, the robot is position controlled to keep the start position (center or periphery), and the subject is visually cued to apply a force toward the direction of the target position (periphery or center—Figure 4B, 1). When the force applied by the subject exceeds a threshold *F_th_* and is sufficient to break through the virtual wall along the desired direction, the controller switches to the second state. In this phase (Figure 4B, 2), the robot is position controlled to reach the target through a minimum-jerk trajectory with duration *t_ST_*. Although subject input is required to trigger the switch to the movement mode, subjects are not involved in controlling their movements during target reaching. The values of *F_th_* are increased on a session-by-session basis, depending on subject ability and comfort (pain and fatigue are recorded before and after each session to ensure excessive levels of each are avoided). This is done to progressively increase the challenge to the subject to encourage active involvement during training.

### 2.5 Outcome Measures

#### 2.5.1 Controller Validation

We analyzed several parameters to evaluate the adaptation of robotic therapy in response to changing patient contribution, both in terms of task assistance and challenge, and in terms of therapy intensity.

To quantify task assistance and challenge in the AAN group, we analyzed the evolution of two controller variables, the feedback control gain and task allotted time, over the therapy program. The feedback controller gain was used as a proxy for the amount of robotic assistance applied during the therapy program, while the allotted time was used as a proxy for task complexity. We analyzed the controller gain values *K_d_* over the duration of each session, calculated for all four DOFs, and averaged for each subject in the AAN group. The change in controller gain value $\Delta K_{d}^{\left(k\right)}$ was obtained for session *k*, for each subject, by subtraction from the average gain at the first training session ${\bar{K}}_{d}^{\left(1\right)}$, i.e., $\Delta K_{d}^{\left(k\right)}={\bar{K}}_{d}^{\left(k\right)}-{\bar{K}}_{d}^{\left(1\right)}$. The changes in feedback gain were then averaged over subjects to obtain the average change in controller gain per session. The allotted time for each session *T*^(^*^k^*^)^ was measured as the allotted time for the last task in each session. Then, as in the controller gain calculation, the change in allotted time Δ*T*^(^*^k^*^)^ relative to Session 1 was calculated and then averaged over subjects per session for all four DOFs.

To quantify how therapy intensity was modulated over time in response to changing patient input, we calculated the change in number of repetitions per session completed during a session Δ*rep*^(^*^k^*^)^. By analyzing the variable Δ*rep*^(^*^k^*^)^ over the therapy program, we could determine the effect of the training on each subject’s capability of performing repeated exercise, which is associated with therapy dose. Finally, for the ST group, we considered the evolution of the force threshold, as percent of a joint-specific maximum value, that the subject was required to apply before triggering the position control mode, a parameter also related to therapy intensity.

#### 2.5.2 Clinical Measures

The primary outcome measure for this study was the Action Research Arm Test (ARAT). The test has a variety of nineteen tasks divided into grasp, pinch grip, and gross arm movement portions. The subject’s motions are graded on a scale of zero to three, with three being a normal motion and zero being an incomplete motion (Lyle, 1981). As secondary outcome measures, the Modified Ashworth Scale (MAS) was used to classify the subject’s spasticity by extending a joint over 1 s. The increase in muscle tone is then rated on a scale from zero (no increase in tone) to four (the affected part or parts are rigid) (Bohannon and Smith, 1987). A third outcome measure was the Grip Pinch Strength assessment, which measures the subject’s pinch and grip strengths using dynamometers, measured in units of force (Kalsi-Ryan et al., 2012). The fourth metric is the Graded Redefined Assessment of Strength, Sensibility and Prehension Test (GRASSP), which measures a subject’s strength, sensation, and prehension in tasks relating to activities of daily life. The test measures a subject’s level of sensation impairment, with zero being no sensation and 4 being the ability to detect 0.4 g of force. The strength measurement is done subjectively by the physical therapist with zero being flaccid and five being a full range with maximal resistance. The prehension portion involves a rating of the ability to grab and maneuver a series of objects on a scale of zero to five with five being the maximal score. The subject is then graded on a scale from zero to four for the ability to grasp a cylindrical object, a lateral key pinch, and a tip to tip pinch (Kalsi-Ryan et al., 2012).

#### 2.5.3 Robotic Measures

Movement kinematics measured during the robotic training and assessment sessions were sampled at 100 Hz for the ST exoskeleton and at 200 Hz for the AAN exoskeleton. Motion data were then processed to extract relevant parameters describing assisted or unperturbed human movements. The raw robotic data were first filtered using a Savitzky–Golay filter with a window length of 21 for the ST exoskeleton and 41 for the AAN exoskeleton. The filter featured different window lengths for the two devices to result in roughly equivalent finite impulse responses in the frequency domain. The data were then passed to a segmentation algorithm to divide the continuous time data into point-to-point segments for data analysis. The segmentation algorithm identified the instants of movement start and movement end by analyzing the regions of subject movement between desired target indicator switches. The algorithm defined *t*_0_ as the time when the desired target indicator changed to initiate subject motion and *t_tar_* as the time when the software acknowledged the subject’s reaching of the desired target. The time of movement start, *t_in_*, was defined as the instant at which the velocity profile exceeded 5% of the peak value for the first time within the target region defined from *t*_0_ to *t_tar_*. The suprathreshold velocity regions were then analyzed to determine their magnitudes, directions in relation to the desired target, and proximity to the previous and subsequent suprathreshold regions. Analysis of suprathreshold regions after *t_tar_* allowed for the inclusion of regions in the movement toward the desired target after the software registers a target reach (i.e., the subject’s correcting for an overshoot of the target). Finally, the movement end, *t_fin_*, was defined as the last time the velocity exceeded 5% of the peak value for the last suprathreshold region corresponding to a movement toward the current desired target. After velocity profile segmentation, metrics of interest were calculated for the cropped time series comprised between *t_in_* and *t_fin_*, to quantify the quality of movement. The metrics used in this study are the mean arrest period ratio (MAPR), spectral arc length (SAL), and normalized speed.

The mean arrest period ratio (MAPR) measures the total amount of time *T_hs_* where the measured velocity is above a predetermined percentage of the peak velocity (Beppu et al., 1984). For this analysis, we used the same threshold used for the definition of movement start (5%) as threshold for the calculation of MAPR. MAPR is then simply defined as $\text{MAPR}=100\frac{T_{\mathrm{hs}}}{t_{\mathrm{fin}}-t_{\mathrm{in}}}$ and defined in the range (0,100]. Aimed movements by healthy individuals would exhibit consistency without peaks and valleys in the velocity profile, leading to a higher MAPR value.

The spectral arc length (SAL) is the negative arc length of the frequency-normalized Fourier magnitude spectrum of the speed profile (Balasubramanian et al., 2012). The metric is defined as

(7) $$\eta =-\int_{0}^{\omega_{c}}\sqrt{{\left(\frac{1}{\omega_{c}}\right)}^{2}+\frac{d\hat{V}\omega^{2}}{d\omega }}d\omega$$

where V(*ω*) is the Fourier magnitude of the speed profile v(t) and [0, *ω_c_* = 10 Hz] is the frequency band of the movement (Fitle et al., 2015). The metric examines the frequency domain of a movement, with the principle that smoother movements have more low frequency components, whereas jerky motions contain more high frequency components. The negative sign is chosen so that a higher value results in a smoother movement.

The normalized speed operates from the observation that healthy movements have fewer valleys and near-stops than an unhealthy motion (Rohrer et al., 2002). This implies that a healthy motion will have a greater normalized mean speed than an impaired motion. The normalized mean speed, or normalized speed, is simply the average speed divided by the maximum speed.

### 2.6 Data Analysis

#### 2.6.1 Controller Validation

The metrics of AAN gain value and allotted time as well as the number of repetitions completed per session are used to measure subject progression over time. To test whether there is a significant change over time, a linear regression was performed on the value of the change per session averaged across all subjects within the group. A 95% confidence interval was generated for the value of the slope.

#### 2.6.2 Clinical Measures

The clinical measures were recorded at the baseline and follow-up sessions (post-treatment, 2-week, and 2-month assessments). There were three cases where the subject did not attend the time-sensitive follow-up sessions (2 weeks post for R11 and 2 weeks and 2 months post for R09) which resulted in an additional 12 incomplete sessions. Thus, all clinical metrics are missing for those assessments. R15 also missed the clinical evaluation in his post-treatment assessment. Furthermore, in 12 of the therapy sessions, the number of repetitions of the therapy portion had to be reduced due to the subject’s late arrival to the session.

The pre–post analysis was performed by comparing the clinical metrics measured during the post-treatment sessions with the value recorded at baseline. The pre-post change in each of the clinical metrics was calculated by subtracting the baseline value from each of the three follow-up values for each subject. Therefore, a value greater than zero would signify an increase in the metric with respect to the baseline, while a negative value would represent a decrease. The changes in the metrics for the pre–post analysis were then tested for statistical significance (*p* < 0.05) via a mixed-design analysis of variance (ANOVA) with treatment group as the between-subjects variable and the DOF, session, and metric variables as the within-subject variables. The Greenhouse-Geisser correction was used when the sphericity assumption was violated. In the event of a significant interaction, the interaction was decomposed using simple main effects. As the analysis technique does not allow missing data, subjects with missing data had to be removed from the analysis. As such, subjects R11 and R15 were only missing data for one of the three follow-up sessions. The missing data for these two subjects were replaced with the subject mean of the other two follow-up sessions for each missing clinical metric. R09 was removed from the analysis, having missed two follow-up sessions. Therefore, the total number of subjects included in the clinical metrics pre–post analysis was 6 and 7 for the AAN and ST groups, respectively.

#### 2.6.3 Robotic Measures

We analyzed movement data acquired during free movements with the robot in the evaluation sessions preceding each therapy session to determine if therapy had an effect on the quality of movements produced by participants. A mixed-design ANOVA was used to analyze the robotic measures collected during the therapy program. Data were grouped by the between-subjects factor (group, with two levels, AAN and ST), and by the within-subject factor (session, with ten levels). The Greenhouse-Geisser correction was used when the sphericity assumption was violated, and significant interactions were decomposed using simple main effects. Due to subject inability to complete the movement or absence from a session, we do not have data for every subject, DOF, and session combination. A complete session is defined as a subject being able to complete an evaluation for a given DOF in a given session. For this study, an average of 87% of sessions were complete. The within-subject completion rate ranged from a maximum of 100% for five different subjects to a minimum of 55% for one subject. Additionally, the within DOF completion rate ranged from a maximum of 97% for the elbow to a minimum of 81% for wrist FE and wrist RUD. The major causes for an incomplete session were the subject being unable to complete an evaluation session of a given DOF due to their level of impairment (10% of all sessions) or a robot hardware failure (1.5% of all sessions). There were eight instances where a subject who began the study unable to complete an evaluation for a particular DOF gained the ability to complete an evaluation before the end of the therapy sessions. Given the multitude of measurements for each subject, we deemed inappropriate to discard data acquired from a given subject due to a few missing data points. Therefore, we established to exclude from the analysis a given subject if data were missing for at least three sessions for that specific subject. Otherwise, we replaced the subject’s missing data with the subject mean. This resulted in the replacement of 16 missing data points (out of a total of 140). The resulting total number of subjects is as follows, represented as (AAN, ST): (7,7) for elbow, (6,6) for wrist PS, (6,5) for wrist FE, and (6,5) for wrist RUD. Because of the rules established for excluding subjects, two subjects were excluded from the analysis for wrist PS, and three subjects were excluded for wrist FE and RUD.

We finally conducted an exploratory analysis to determine whether the effect of the training program in the two groups was captured by a linear increase over session of the robotic outcome measures. For this analysis, a change in metric is defined for each therapy session *i* as the difference between the outcome measure obtained in session *i* and the metric obtained in the first training session for which the subject has data. As such, the baseline is taken as the first time the subject is able to perform the motion, and the change is calculated relative to this baseline throughout the duration of the therapy sessions. This approach appears suitable to describe within-subject changes in outcome measures, as it avoids confounds associated with data replacement with the mean as done for the mixed-design ANOVA; however, this approach creates unbalanced groups in both the between-subject and within-subject factors. To test significance of the effect of the within-subject repeated measure (i.e., session), a linear regression was conducted on the change in robotic metric averaged across all subjects within a group. This was accomplished by two separate regression analyses, one for the AAN group, and one for the ST group.

## 3 Results

We show characteristics of our controller behavior as recorded during a parallel-group balanced controlled trial with subjects with incomplete SCI and compare performance differences across our two treatment groups. We start by describing the behavior of our AAN and ST controllers over each session of the study to elucidate how each controller modulates intensity of treatment. Then, we evaluate changes in subject capability as measured by standard clinical assessments. Finally, we quantify longitudinal changes in subjects’ movement quality using measurements provided by evaluation sessions conducted during the therapy program.

### 3.1 Validation of the AAN and ST Controllers

The AAN controller is designed to modulate both the amount of assistance and challenge for reaching tasks in an automated way, based on the performance of the subject. The behavior of the ST controller can be modulated manually by the therapist by adjusting the threshold level to increase or decrease challenge on a session-by-session basis. As such, different metrics were used to test how the two controllers modulated assistance, challenge, and therapy intensity. In validation of both controllers, no adverse events (i.e., injury or excessive fatigue reported by subjects) were reported in this study during the therapy sessions.

#### 3.1.1 Task Assistance and Challenge

**Figure 5:**
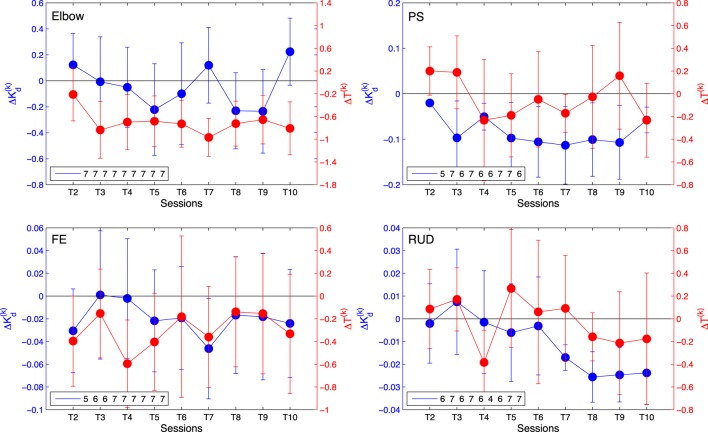
Change in average controller gain $\Delta K_{\text{d}}^{\left(k\right)}$ (blue) and allotted time Δ*T*^(^*^k^*^)^ (red) per session relative to session T1 for elbow [upper left], wrist PS [upper right], wrist FE [lower left], and wrist RUD [lower right]. Negative values indicate a decrease in the amount of assistance (gain) received or amount of time allotted for the task, respectively. The legend indicates the number of AAN subjects who completed the task at each training session. Error bars extend to ± the standard error for the group.

Via the linear regression analysis, we determined that the controller gain is significantly decreased over therapy sessions in all DOFs with the exception of the elbow joint (Figure 5). This demonstrates that the amount of assistance applied by the controller, expressed by the dynamics of its feedback controller gain, was decreased through the therapy program. The slope estimates, expressed as mean ± standard deviation, are −0.007 ± 0.01 for the elbow, −0.013 ± 0.002 for wrist PS, −0.003 ± 0.001 for wrist FE, and −0.0021 ± 0.0005 for wrist RUD.

Via the linear regression analysis, we also determined that allocated time for task completion decreases significantly over the course of the therapy program in several joints, and it did not increase in any joint. The slope estimates, expressed as mean ± standard error, are −0.10 ± 0.02 for the elbow, −0.009 ± 0.01 for wrist PS, −0.04 ± 0.01 for wrist FE, and −0.009 ± 0.01 for wrist RUD. The slope estimate intervals indicate that the decreasing trend in change in allocated time is significant at the *p* < 0.05 confidence level for the elbow and wrist FE DOFs, which demonstrates that for those joints, the challenge offered by therapy sessions, measured by allocated time, significantly increased over the duration of the therapy program.

#### 3.1.2 Therapy Intensity

**Figure 6:**
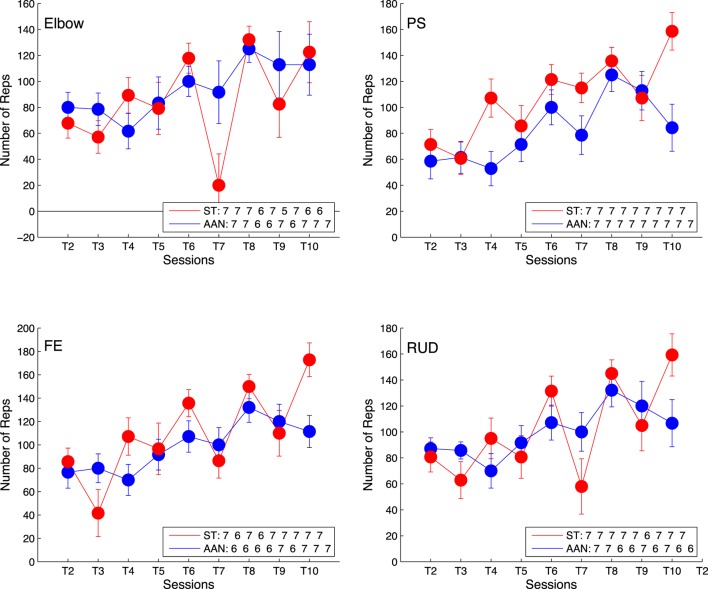
Comparison of number of training repetitions completed per session relative to training session T1 for elbow [upper left], wrist PS [upper right], wrist FE [lower left], and wrist RUD [lower right]. The legend indicates the number of subjects who completed the task at each training session. Error bars extend to ± the standard error for the group.

The number of completed repetitions, averaged across all subjects for each session, were summed and are displayed in Figure 6. The plot represents the difference in number of repetitions completed with respect to session 1, such that an increasing trend indicates a sustained change in completed repetitions from session to session. Via the linear regression analysis, we demonstrated that the slope of the measure of number of repetitions completed per each session is greater than zero at the *p* < 0.05 confidence level for all DOFs in both the AAN and ST groups. The slope estimates, expressed as mean ± standard error, for the ST group are 12.8 ± 2.4 for the elbow, 16.5 ± 1.6 for wrist PS, 17.0 ± 2.0 for wrist FE, and15.7 ± 2.1 for wrist RUD. The slopes for the AAN group are 14.2 ± 1.6 for the elbow, 12.7 ± 1.5 for wrist PS, 14.9 ± 1.6 for wrist FEF, and 14.9 ± 1.9 for wrist RUD. Based on these estimates, it can be concluded that the number of repetitions per sessions increased for both the AAN and ST groups.

**Figure 7:**
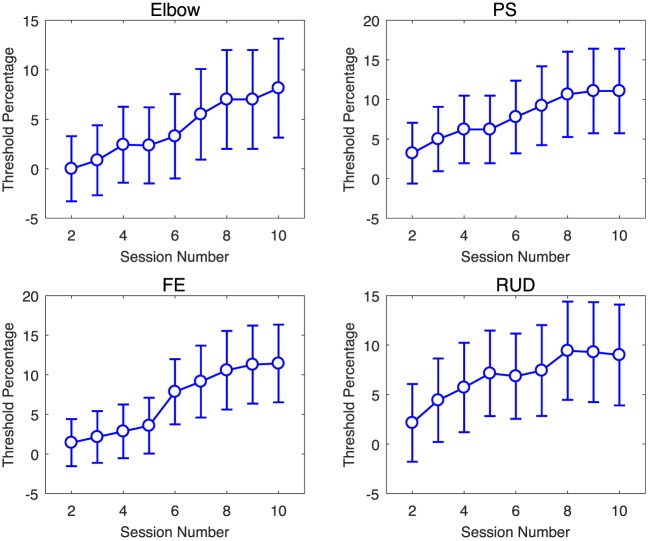
Percent change in ST group force threshold during the therapy program, relative the value used in the first session. Error bars extend to ± the standard error for the group.

For the ST group, an additional parameter that was adjusted to modulate therapy intensity was the force threshold *F_th_*, which was adjusted on a session-by-session basis depending on subject ability and comfort on the previous sessions. The percent change in force threshold *F_th_*, calculated relative to the value used for the first session, increased for all joints during the therapy program, as shown by Figure 7.

### 3.2 Clinical Measures

**Figure 8:**
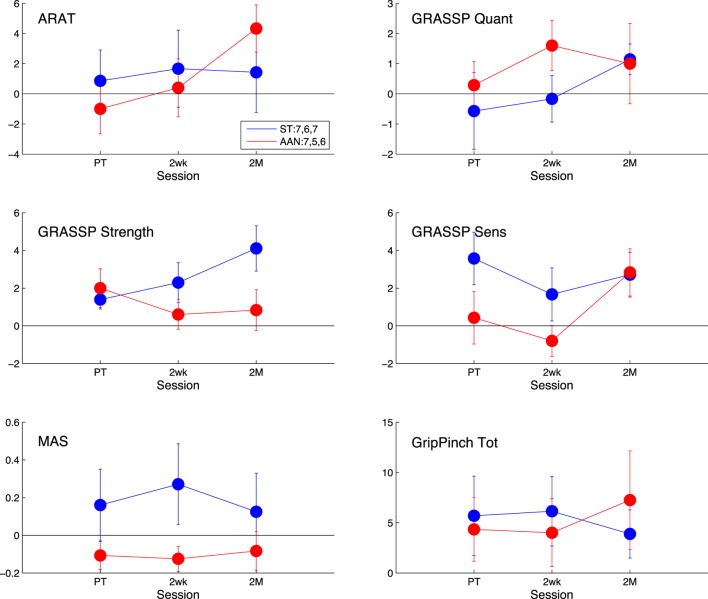
Comparison of the clinical measures to baseline, measured post-treatment (PT), 2 weeks after treatment (2wk), and 2 months after treatment (2 M). The AAN values are shown in red, and the ST values are shown in blue. The clinical measures presented are the Action Research Arm Test (ARAT) [upper left], the quantitative [upper right], strength [middle-left], and sensation [middle-right] portions of the Graded Redefined Assessment of Strength, Sensibility, and Prehension Test (GRASSP), the Modified Ashworth Scale (MAS) [lower left], and the Grip Pinch Strength assessment [lower right]. The legend indicates the number of subjects who completed the task at each session for that measure. Error bars extend to ± the standard error for the group.

No significant effect of the within-subject factor (session) was observed for the primary outcome measure, i.e., the change in ARAT score (*p* = 0.128). As such, the null hypothesis of this study is not falsified. The results of the mixed-design ANOVA are presented in Table 3, while the evolution of the subject-by-subject change in each metric is reported in Figure 8 as a difference relative to the pre-treatment measurement. Some of the secondary outcome measures selected for this study, namely, the GRASSP Strength and GRASSP Sensation metrics showed a significant result, although the result has not been corrected for multiple comparisons. No significant interactions, including the effect of the between-subject variable (experimental group), were measured neither in the primary outcome measure nor in other clinical measures.

**Table 3:** ANOVA results for clinical measures.

Metric	df	F_session_	p_session_	F_group⋅session_	p_group⋅session_
ARAT	(3, 33)	2.04	0.128	1.175	0.334
GRASSP Quant	(3, 33)	1.44	0.250	0.467	0.707
GRASSP Strength	(3, 33)	3.35	**0.031**	2.663	0.064
GRASSP Sens	(3, 33)	6.42	**0.002**	0.642	0.594
GripPinch	(3, 33)	3.24	0.079	1.943	0184
MAS	(3, 33)	0.18	0.752	0.697	0.467

*N = 6 for AAN and N = 7 for ST for all metrics*.

*Significance values are included under column “p.” Values are in bold if they are significant at p < 0.05. The chosen significance level is reported in the Materials and Methods section*.

### 3.3 Robotic Measures

Via the mixed-design ANOVA, we quantified the longitudinal evolution of robotic measures of quality of movement over training sessions in both groups. For the metric SAL, a significant effect of the factor session was measured in the elbow and wrist RUD joint. For the metric MAPR, wrist FE and wrist RUD showed a significant effect of session. For normalized speed, wrist PS and wrist RUD showed a significant effect of session. The results of the mixed-design ANOVA are included in Table 4.

**Table 4:** ANOVA results for robotic measures: therapy sessions.

DOF	Metric	df	F_session_	p_session_	F_group⋅session_	p_group⋅session_
Elbow	Norm speed	(9, 108)	2.43	0.062	0.95	0.444
	MAPR	(9, 108)	2.22	0.076	1.34	0.265
	SAL	(9, 108)	3.22	**0.034**	2.75	0.058
PS	Norm speed	(9, 90)	2.63	**0.009**	1.02	0.428
	MAPR	(9, 90)	2.12	0.122	1.23	0.318
	SAL	(9, 90)	1.34	0.277	0.53	0.678
FE	Norm speed	(9, 81)	1.94	0.151	1.16	0.341
	MAPR	(9, 81)	2.27	**0.025**	0.73	0.683
	SAL	(9, 81)	2.36	0.112	0.85	0.456
RUD	Norm speed	(9, 81)	2.72	**0.008**	3.01	**0.004**
	MAPR	(9, 81)	1.95	0.057	2.49	**0.015**
	SAL	(9, 81)	2.15	0.124	3.73	**0.027**

*N = (AAN, ST): N = (7, 7) for elbow. N = (6, 6) for wrist PS*.

*N = (6, 5) for wrist FE. N = (6, 5) for wrist RUD*.

*Significance values are included under column “p.” Values are in bold if they are significant at p < 0.05. The chosen significance level is reported in the Materials and Methods section*.

All three metrics exhibited significant interactions for wrist RUD: (*F*(9, 81) = 3.01, *p* = 0.004) for normalized speed, (*F*(9, 81) = 2.49, *p* = 0.015) for MAPR, and (*F*(9, 81) = 3.73, *p* = 0.027) for SAL. These interactions were decomposed using simple main effects to reveal that only the AAN group exhibited a significant improvement in all of these metrics for wrist RUD. The AAN and ST results were (*F*(9, 36) = 5.09, *p* < 0.001) and (*F*(9, 36) = 1.33, *p* = 0.256) for normalized speed, (*F*(9, 36) = 3.39, *p* = 0.003) and (*F*(9, 36) = 0.96, *p* = 0.488) for MAPR, and (*F*(9, 36) = 4.04, *p* = 0.001) and (*F*(9, 36) = 1.16, *p* = 0.352) for SAL, respectively. These results demonstrate both an overall positive effect of the treatment on the outcome measure measured on a session-by-session basis, and a differential effect of the experimental group (i.e., AAN or ST). Analysis of the robotic measures provides results in contrast to those deriving from clinical measures.

**Figure 9:**
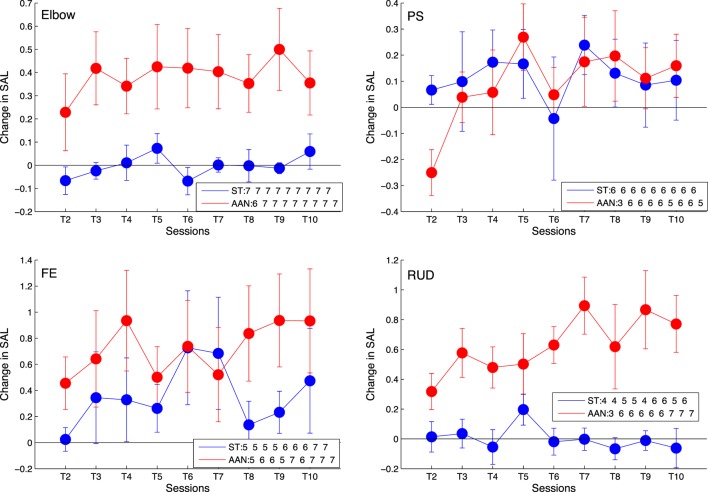
Longitudinal outcomes for spectral arc length (SAL) showing change in metric for each training session relative to training session T1 for elbow [upper left], wrist PS [upper right], wrist FE [lower left], and wrist RUD [lower right]. Positive values indicate smoother movements than exhibited in T1. Linearly increasing trends indicate continuous improvement in movement smoothness during the course of therapy. The legend indicates the number of subjects who completed the task at each training session. Error bars extend to ± the standard error for the group.

The session-by-session changes in robotic measures can be visualized in the training session plots, presented in Figure 9 for the SAL metric, and in Figures S1 and S2 in Supplementary Material for the other robotic measures extracted from the data (MAPR and normalized speed, respectively). The plots across each training session provide a more detailed representation of the actual progression made by each group to independently move the robotic device in each DOF, evaluated on the exoskeleton used during their training. Best fitting regression lines describing the change over session in robotic metrics were calculated for each group, and the corresponding slopes were shown to be significantly different from zero at the *p* < 0.05 level in all joints and metrics for the AAN group, while only in 5/16 cases for the ST group. The entire set of estimated slopes and associated confidence interval are displayed in Table 5. Bolded values in the table indicate that the regression slope is positive at *p* < 0.05.

**Table 5:** Linear regression slope and confidence interval for robotic metrics.

	Normalized speed [1/session]		MAPR [%/session]		SAL [1/session]	
	AAN	ST	AAN	ST	AAN	ST
Elbow	**0.0052 ***±*** 0.0007**	0.00071 ± 0.0011	**0.57 ***±*** 0.086**	0.058 ± 0.071	**0.063 ***±*** 0.012**	0.00102 ± 0.0033
PS	**0.0053 ***±*** 0.00065**	**0.0018 ***±*** 0.0017**	**0.166 ***±*** 0.068**	0.11 ± 0.14	**0.021 ***±*** 0.0085**	**0.018 ***±*** 0.0066**
FE	**0.0078 ***±*** 0.0009**	0.00046** ± **0.0011	**0.823 ***±*** 0.12**	**0.25 ***±*** 0.14**	**0.12 ***±*** 0.020**	**0.062 ***±*** 0.018**
RUD	**0.0086 ***±*** 0.0012**	−0.00033 ± 0.00144	**0.664 ***±*** 0.13**	**0.204 ***±*** 0.1324**	**0.11 ***±*** 0.014**	−0.0018** ± **0.0055

## 4 Discussion

This paper presented a parallel-group, controlled trial (PGCT) to evaluate the effects of assist-as-needed (AAN) assistance in robot-aided neurorehabilitation after incomplete spinal cord injury (iSCI). The study compared the effects of AAN treatment with those provided by an alternative intervention, subject-triggered (ST) control, matched in terms of total therapy time. We present for the first time validation of the AAN robotic controller in subjects with iSCI and demonstrate feasibility and consistency of controller performance over a 10-session period with this clinical population. As far as the clinical results are concerned, difficulties in the recruitment of the identified population (patients with iSCI affecting upper extremity function available to participate in a 3-month long rehabilitation program) prevented achievement of the sample size that had been identified to detect a significant effect in the clinical primary outcome measure (i.e., pre–post ARAT score being greater in the AAN group relative to the control group). As a result, the null hypothesis of this clinical study could not be rejected. At the same time, kinematic data measured during evaluation sessions during the therapy program provide support for the hypothesis that improvement in quality of movement was achieved in both groups, with the AAN group showing larger improvements in smoothness metrics, compared to the control ST group. While the differential effect of the therapy program on robotic measurements was demonstrated quantitatively only in one of the four joints treated (wrist RUD), an exploratory analysis showed that the slope of the linear change in outcome measure over sessions was consistently greater in the AAN group than in the ST group. The following section will discuss in more detail the results obtained in this study.

### 4.1 Validation of the AAN and ST Controller

#### 4.1.1 Task Assistance and Challenge

The AAN controller can modulate both task assistance and challenge continuously during robot-assisted therapy. Task assistance and challenge were quantified for the AAN controller by the feedback control gain *K_D_* and allocated time *T*, whose change over time relative to session 1 are represented in Figure 5. While the change in gains Δ*K_D_* for the elbow DOF are relatively constant over the course of sessions, the other three DOFs show a decrease in value, with wrist PS having the largest decrease over time. The decrease in gain values with respect to the baseline signifies the reduced assistance from the controller over time. Thus, the negative trend of controller gains over sessions implies that the subjects were more capable of completing the movements as the study progressed. Conversely, the average changes in allotted time Δ*T*^(^*^k^*^)^ show the largest decrease for the elbow. Wrist FE also exhibited a slight decrease over time, whereas wrist PS and wrist RUD remain relatively stable. When comparing controller gains and the allotted time, we see that for some DOFs it is the amount of assistance (via a reduction in feedback gain) that varies, while for other DOFs the controller performance variations are dominated by reductions in allotted time. Reductions in the gain metric and in the allotted time over the course of the study both demonstrate an increase in the subject’s ability to perform the movement and show the responsiveness of the AAN controller to this performance improvement, with a resulting increase in task complexity, thereby keeping “challenge” at constant levels (Zimmerli et al., 2012).

Regression analysis of either controller gain or allocated time show statistically significant effect of session in all DOFs. For the wrist FE DOF, the effect is significant for both control gain, and allocated time metrics. For the elbow, the gain slope included zero in the confidence interval, and for PS and wrist RUD, the allotted time slope confidence interval included zero. Since both allocated time and gain combine to modulate the task difficulty, and at least one of the two parameters is significantly altered by session for all DOFs, this analysis supports the role of the AAN in modulating task assistance and challenge in response to growing patient input.

These findings are well aligned with our prior demonstrations of the assist-as-needed controller where healthy subjects were asked to modulate their compliance with the controller action and their movement speed to illustrate the behavior of gain and allotted time modulation algorithms (Pehlivan et al., 2015). In the current study, a similar behavior is observed in this neurologically impaired population.

#### 4.1.2 Therapy Intensity

From Figure 6, there is an observable increase in the number of repetitions from training session T1 to T10 for both the AAN and ST groups. For the ST group, the therapist is encouraging faster movements and shorter pauses between movements, resulting in an increase in intensity throughout the therapy protocol. Similarly, the AAN controller is modulating the assistance (via feedback gain) and the allotted time, resulting in more movements completed in each session. Both controllers successfully facilitate the increase of therapy intensity via increased repetitions. There is some variability between sessions as several factors combine to affect the number of repetitions able to be completed. Additionally, subjects were undergoing multiple trainings per week, so they might be fatigued or stiff on any given day, which would diminish the number of reps they could complete on a given day. It is worth noting that data included in Figure 6 represents the change in number of repetitions with respect to the baseline, thus a positive value represents an increase in the number of repetitions completed in a session relative to the first session. Even with the variability between sessions, all values are positive, which represents an increase in repetitions compared to their baseline behavior. This finding suggests that via the training program, both through the ST and AAN controllers, the subjects are prompted to complete more repetitions per session.

In addition to the number of movement repetitions, another parameter that is associated with the intensity of the therapy program is the force threshold *F_th_* that the subject is required to produce for each repetition, in the ST control group. Also this metric was shown to be increasing over the therapy program for subjects in the ST group, further confirming that therapy intensity was gradually increased on a session-by-session basis in the ST group.

### 4.2 Clinical Measures

Clinical assessments were conducted prior to the start of the therapy protocol, then at the conclusion of the therapy sessions. Retention was assessed by conducting these assessments again at 2 weeks and 2 months post-treatment. The impact of the robotic rehabilitation intervention can be evaluated by comparing changes in these metrics from pre- to post-treatment, and also by analyzing the retention at follow-up assessments.

No significant effect of session was extracted in the analysis of the effect of session in the primary outcome measure, i.e., the ARAT score, nor of the interaction between session and group. From analysis of the ARAT clinical metric at each session, it can be seen that the ST group shows an increase of roughly one point in ARAT score with respect to the baseline at the post-therapy time point, which is sustained in the subsequent follow-ups. Alternatively, the ARAT score in the AAN group initially decreases, while later increasing to an average change in ARAT score of 4.33 points at the 2-month mark, a gain that is greater than that of the ST group. Due to the subjective nature of the clinical assessments, minimally clinically important differences (MCID) are introduced to define a clinically significant increase in a metric. MCIDs attempt to account for variability from test–retest and inter-rater reliability effects. In stroke, the MCID for ARAT is 5.7 points (van der Lee et al., 2001), while it is not established for iSCI. Thus, the observed increase in the AAN group is likely to not be clinically significant.

The GRASSP Strength and GRASSP Sens metrics were the only two metrics showing a statistically significant effect of session at (*p* = 0.031 and *p* = 0.002, respectively); however, no significant interaction between group and session was measured. The significant increase in the GRASSP Strength metric was expected, as repetitive use over time of muscles should increase their strength. The increase in the GRASSP Sens metric, however, was unanticipated, as we are not focusing any of the training efforts on increasing the subject’s touch perception as a part of the robotic therapy. A possible explanation would be that the forced repetitions caused the subject to engage their arms more than they were used to, which resulted in more familiarity with the arm and thus a heightened sense of perception. As the GRASSP Sens metric was not considered as a primary outcome measure, further research is necessary to draw any conclusions from this finding.

Changes in MAS relative to baseline are relatively small; neither group showed any meaningful change at the follow-up sessions with respect to the baseline. The AAN group had a decrease in MAS score of 0.11, 0.13, and 0.08 for the post-treatment, 2-week, and 2- month follow-up, respectively, whereas the ST group demonstrated an increase of 0.16, 0.27, and 0.13. The MCID for MAS has not been established yet. However, given that the MAS scale ranges from 0 to 4 and given that in a comparable study in stroke the minimal detectable change was 1 point (Shaw et al., 2010), these small differences in the pre–post analysis do not indicate a meaningful change in the metric over time.

Finally, we observe that both groups increase their grip and pinch score relative to baseline, and that the increased score is sustained in subsequent follow-up visits. The ST group begins at 25.3 N and is relatively constant until the 2-month follow-up, where it decreases to a relative measurement of 17.3 N. The AAN group is relatively stable with an improvement of 17.8 N from the post-treatment to the 2-week follow-up and then increases to 32.2 N at the 2-month follow-up.

### 4.3 Robotic Measures

A richer insight into the impact of robotic controllers on movement quality is provided by the longitudinal analysis of movement quality data along the therapy program, as provided by the robotic metrics SAL, MAPR, and normalized speed. Both groups exhibit an increase in the robotic metrics over the course of the therapy program, as visible from Figure 9, although the significance of the factor session in the robotic metric ANOVA varies within a given joint depending on the specific metric considered. A significant interaction between group and session was measured only for wrist RUD movements. From the longitudinal analysis of robotic evaluation data, it can be observed that the AAN group showed significant improvement in all DOFs, while the ST group showed statistically significant improvement in only wrist PS and wrist FE (Figure 9).

Analysis of repeated measurements obtained during the therapy program illustrate fluctuations in the observed movement smoothness, which illustrate how subjects can perform differently depending on fatigue or other factors from one day to the next. These fluctuations could also be occurring in the baseline and follow-up analysis, making pre–post comparisons insensitive to trends that can only be observed through the longitudinal analysis. By comparing all of the therapy sessions, we have many more data points which allow for a general trend to be observed with diminished influence of day-to-day variations in performance. These observations are really only feasible if using assessments that can be gathered as part of the therapy protocol, such as via the evaluation trials that we incorporated into this study design, and computed with readily available data. It is impractical to conduct clinical assessments such as ARAT at every training session due to the time constraints of typical therapy sessions. This observation supports the value of robotic measures of movement coordination as a practical tool useful for evaluation of recovery of motor function during robot-aided therapy.

## 5 Conclusion

This paper presents the results of a parallel-group controlled trial (PGCT) to test the efficacy of a novel AAN controller in robotic rehabilitation after incomplete spinal cord injury. With its design features (presence of an active control condition, blindness of the evaluator to treatment assignment, and execution of a power analysis for the primary study outcomes), this study falls within the category of stage 2, *development-of-concept* pilot studies, despite the relatively small sample size emerging as a result of the power analysis (*N* = 20). As such, to the best of our knowledge, this is the first time this type of study has been conducted in the field of robot-assisted therapy for upper extremity rehabilitation in incomplete spinal cord injury.

We present details on the methods of our study, including thorough descriptions of the controller modes and treatment regimens implemented on the MAHI Exo-II upper limb exoskeleton robot. We have introduced methodological features in the study design which are of interest to the rehabilitation robotics community. In particular, the presented scheme of sequential group assignment with covariates minimization guarantees the desired level of balance of covariates in the two groups, a feature that cannot be reliably achieved with unrestricted randomization in studies with low (*N* < 50) sample sizes (Schulz and Grimes, 2002).

The results presented in this paper highlight its two major contributions. First, we presented data to validate the operation of our assist-as-needed (AAN) robotic controller to adjust controller gains and allotted times for movement completion to modulate the challenge and assistance provided to the subject in an automated fashion. The automated nature of assistance modulation via gain adjustment and challenge modulation via changing of the allotted time for movement completion were comparable to the progression of challenge achieved manually with the subject-triggered (ST) controller. With the ST control approach, the therapist adjusted challenge of treatment delivery by manually controlling the force threshold for initiating movement via a GUI, and challenge via coaching and encouragement to elicit faster movements. The results demonstrate for the first time in an impaired population the modulation of AAN controller action in response to subject performance throughout a therapy regimen.

Our second contribution involves the analysis of the differential effects of a novel controller for robot-aided rehabilitation therapy on patients affected by iSCI. This analysis has been conducted using both clinical metrics (collected at baseline, posttreatment, and at two follow-up sessions) and robotic metrics (collected longitudinally during the therapy program). Only weak gains were observed in the clinical outcome measures, with no support for either controller showing a clinically nor statistically significant increase in clinical metrics. While some improvements (such as with the GRASSP metric) were statistically significant, the observed gains failed to translate into clinically meaningful findings. Despite this weak result, longitudinal analysis of robotic measures shed light on the session-by-session changes in subject performance in terms of the movement quality metrics derived from robot kinematic data. The AAN group consistently showed improvement in performance across all DOFs and all robotic measures of movement quality, while the ST group showed smaller gains confined to only a subset of the metrics and DOFs. Based on these findings, further research is warranted to evaluate the potential of AAN control strategies for robotic rehabilitation of the upper limb following incomplete SCI. Given the continually improving performance of the AAN group in our study, therapy protocols incorporating a greater number of therapy sessions may achieve minimally clinically significant differences in clinical outcomes that we were unable to demonstrate in this study.

## Availability of Data and Supporting Material

Data will be made available upon request to scientist interested in secondary analyses.

## Ethics Statement

This study was reviewed and approved by the institutional review boards (IRB) of Rice University and our clinical collaborators’ institutions (Rice University IRB 654451, UT Health/TIRR Memorial Hermann IRB HSC-GEN-13-0315), with written informed consent from all subjects. All subjects gave written informed consent in accordance with the Declaration of Helsinki. This study has been retrospectively registered on Clinicaltrials.gov, registration number: NCT02803255.

## Author Contributions

JF collected data, conducted data analysis and statistical testing, and wrote the manuscript. JE collected data and conducted data analysis, and wrote the manuscript. AP implemented the AAN controller. KF collected data and performed preliminary data analysis. KN performed the evaluations. GF provided clinical supervision regarding inclusion/exclusion criteria and presentation of clinical data. FS conceived and designed the study, obtained funding, supported controller development, data analysis, and statistical testing, and wrote the manuscript. MO designed the study, obtained funding, supported controller development, data analysis, and statistical testing, and wrote the manuscript.

## Conflict of Interest Statement

The authors declare that the research was conducted in the absence of any commercial or financial relationships that could be construed as a potential conflict of interest.

## Footnotes

^1^Prevalence of incomplete SCI is roughly 0.1% of the population, whereas in stroke it is 2.9% (Go et al., 2014).

^2^The software STPLAN, University of Texas M. D. Anderson Cancer Center, Houston, TX, was used for the power analysis.

## Abbreviations

SCI, spinal cord injury; AAN, assist-as-needed; MAHI, mechatronics and haptic interfaces; PCGT, parallel-group controlled trial; ST, subject-triggered; ASIA, American Spinal Cord Injury Association; MAS, Modified Ashworth Scale; DOF, degree-of-freedom; RBF, Radial Basis Function; CTR, conditional trajectory recalculation; GRASSP, Graded Redefined Assessment of Strength, Sensibility and Prehension Test; MAPR, mean arrest period ratio; ANOVA, analysis of variance; SAL, spectral arc length; FE, flexion/extension; RUD, radial/ulnar deviation; PS, pronation/supination; MCID, minimally clinically important difference.
