# Supplementary figures and images for: Effects of Assist-As-Needed Upper Extremity Robotic Therapy after Incomplete Spinal Cord Injury: A Parallel-Group Controlled Trial
Source: Front Neurorobot. 2017 Jun 13;11:26. doi: 10.3389/fnbot.2017.00026 (PMC5469353; doi:10.3389/fnbot.2017.00026)

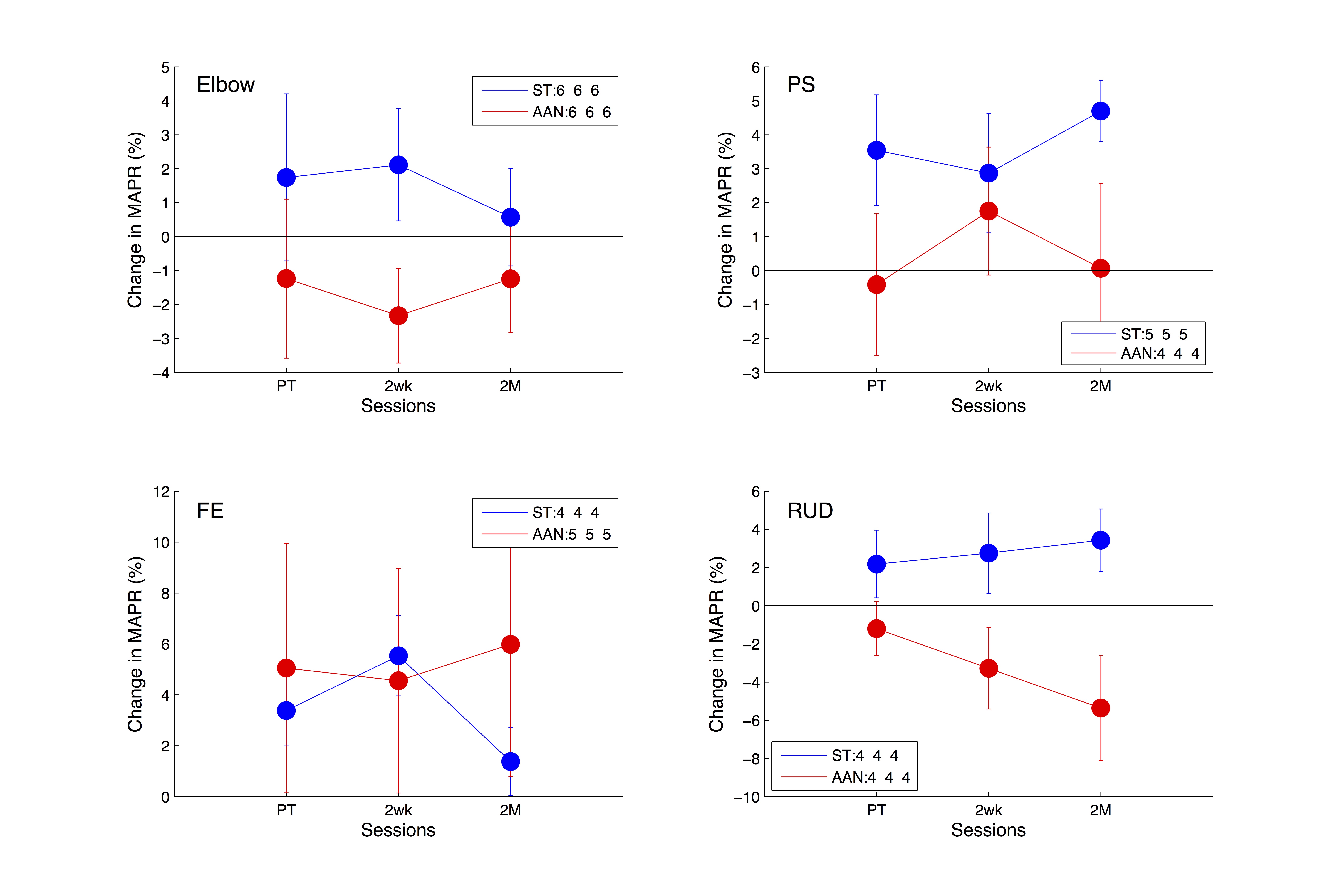

Supplement: Supplementary file 1 [file image_1.jpeg]

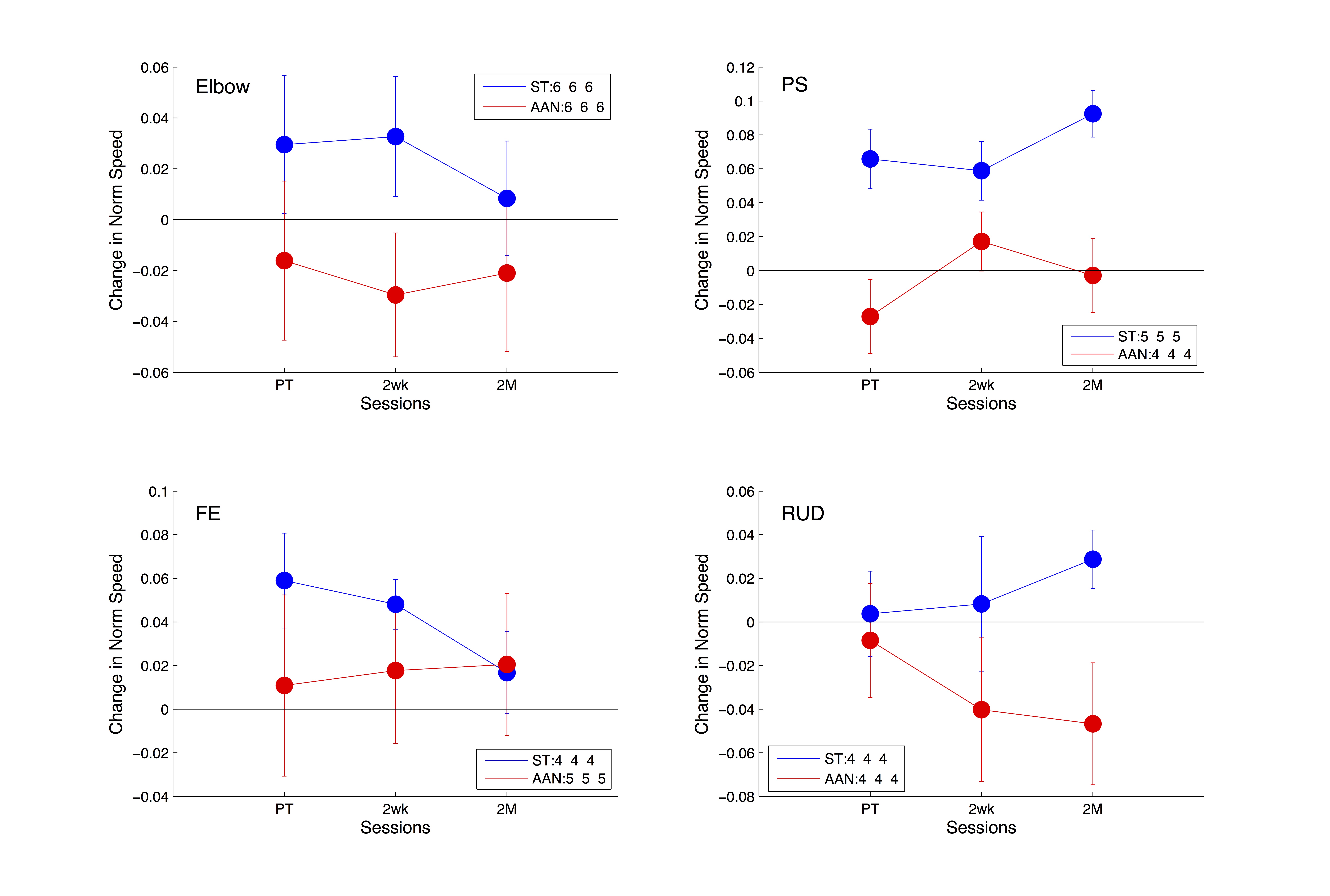

Supplement: Supplementary file 2 [file image_2.jpeg]
